# A modular, reusable biocatalytic flow system for UDP-GlcNAc production†

**DOI:** 10.1039/d5re00127g

**Published:** 2025-05-09

**Authors:** Tom L. Roberts, Jonathan P. Dolan, Gavin J. Miller, Marcelo A. D. Lima, Sebastian C. Cosgrove

**Affiliations:** a Lennard-Jones Laboratory, School of Chemical & Physical Sciences, Keele University Keele Staffordshire ST5 5BG UK g.j.miller@keele.ac.uk s.cosgrove@keele.ac.uk; b Centre for Glycoscience, Keele University Keele Staffordshire ST5 5BG UK m.andrade.de.lima@keele.ac.uk; c School of Life Sciences, Keele University Keele Staffordshire ST5 5BG UK

## Abstract

We report here the continuous flow synthesis of a high-value sugar nucleotide. Immobilisation of enzymes onto solid carriers permitted transfer of the biocatalysts into packed bed reactors to realise a continuous biocatalytic platform for the synthesis of uridine diphosphate *N*-acetylglucosamine (UDP-GlcNAc) on 100 mg scale, with capacity for multiple reuses. The modular continuous flow approach described here represents a significant, up to 11-fold, improvement in space time yield (STY) when compared to batch studies, along with preventing product induced enzyme inhibition, reducing the need for an additional enzyme to break down inorganic pyrophosphate (PPi). The modular nature of the system has also allowed tailored conditions to be applied to each enzyme, overcoming issues relating to thermal stability. This development presents a platform approach towards a more efficient, continuous synthesis of important glycan targets including glycoproteins, specific oligosaccharide sequences and glycosylated drug targets.

## Abstract 2

Roberts *et al.* demonstrate UDP-*N*-acetyl glucosamine can be produced in a continuous biocatalytic system. Improvements *versus* batch include simplified downstream processing, 11-fold increase in space time yield and improvement in enzyme lifetime.

## Graphical abstract

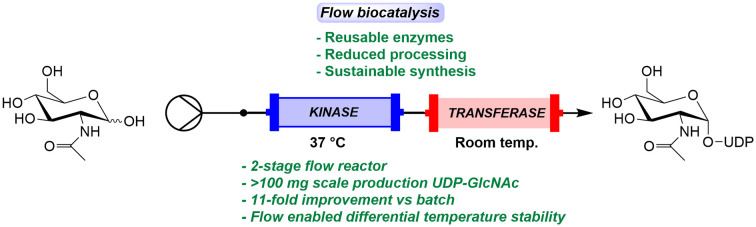

## Introduction

With the spotlight increasingly shone on sustainability in recent years, biocatalysis has emerged as an alternative way to synthesise key intermediates for natural products and bioactive molecules.^1^ A reduction in the number of steps required, less side product formation, and the avoidance of toxic solvents are quoted as key advantages over chemical synthesis with biocatalytic transformations representing potentially shorter routes to some key products. This can be exemplified by the large growth in research centred around multi-enzyme cascade reactions.^2,3^ For large scale applications though, the cost of enzyme production coupled with poor recoverability means soluble enzymes are often overlooked in favour of chemical alternatives.^4^ Therefore, to further the use of biocatalysis, it is necessary to create stable biocatalysts that can be reused multiple times.^5^ Enzyme immobilisation is a growing discipline and provides a mechanism for increased reusability.^6,7^ Their stability can be enhanced by immobilisation, although sometimes at the cost of lower activity.^8,9^ Immobilised enzymes can also be incorporated into continuous flow systems,^10–12^ for example in packed bed reactors.^13–15^ Specifically, this can be used to apply separate conditions to multiple enzymes in one cascade with differential reaction needs (*i.e.*, temperature or pH stability).^16^ Despite industrial chemical synthesis typically being performed in batch, the pharmaceutical industry has filed several patents that use flow in recent years.^17^ This higher uptake of continuous processes coupled with the expanding synthetic scope of enzymes means flow biocatalysis now offers a viable option for numerous synthetic applications.

**Fig. 1:**
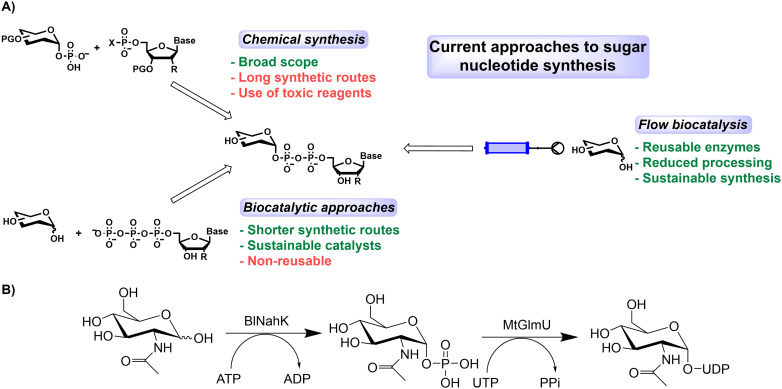
A) Current approaches to sugar nucleotide synthesis and B) biotransformation of GlcNAc to UDP-GlcNAc utilising two enzymes, BlNahK and MtGlmU. In soluble batch cascades a further enzyme (iPPase) is added to break down the inorganic phosphate (PPi) which has an inhibitory effect on the MtGlmU.^30^

Despite the rapid development of biocatalysis, carbohydrate bioprocess development has somewhat lagged behind, which is surprising due to the complexity of traditional chemical synthesis of carbohydrate targets.^18^ Carbohydrate building blocks, synthesised chemically or biocatalytically,^18,19^ are essential for several applications including glycan synthesis,^20^ and *in vitro* post-translational modification of proteins.^21,22^ Additionally, a significant number of approved therapeutic proteins, including eight of the top-ten selling biologics in the 2010s, are glycoproteins.^23^ Small molecule drugs containing sugars have also recently been approved by the FDA, such as dapagliflozin, used in the treatment of type 2 diabetes mellitus (T2DM).^24^ The overall number of glycosylated small molecule drugs, however, remains low (nine out of 200 approved between 2015–2020).^25^ Sugar nucleotides are key building blocks for enzymatic glycosylation however current approaches to their synthesis suffer some notable disadvantages (Fig. 1A). (Chemo)enzymatic syntheses have been reported for both natural and non-natural analogues,^26–28^ so improved access to different sugar nucleotides is essential to improve the synthesis of both existing and novel glycosylated synthetic targets. Amongst the most important is UDP-GlcNAc, which is key for several important applications, including many of those listed above. This sugar nucleotide has seen many innovative methods utilised for its enzymatic synthesis, making using of a sugar kinase (Nahk) and then a Uridyltransferase (either AGX1 or GlmU).^29–33^ This biological importance underlines the need to access the nucleotide derivative in usable quantities.^20–22^

Herein, we describe the optimisation of a flow system to realise the continuous biocatalytic synthesis of UDP-GlcNAc. The use of a modular flow system was essential due to thermal incompatibility between the required enzymes, and it permitted 100 mg quantities of the sugar nucleotide to be isolated from multiple, sequential reactions using the same immobilised bioreactor.

## Results and discussion

### Initial batch testing

UDP-GlcNAc is synthesised enzymatically from *N*-acetylglucosamine through a kinase mediated 1-OH phosphorylation, which is then converted to UDP-GlcNAc *via* a transferase (Fig. 1B). The enzymes chosen for screening (Table S1†) were the kinase from *Bifidobacterium longum* (BlNahK) and the uridyltransferase from *Mycobacterium tuberculosis* (MtGlmU). A series of carriers for enzyme immobilisation were tested for their potential to facilitate the immobilisation of BlNahK and MtGlmU. These included an amino carrier (ECR8309F) to which the enzyme is covalently bound in a nonselective manner *via* a glutaraldehyde cross-linker (Fig. S1†), and a series of EziG carriers which selectively coordinate *via* an Fe^3+^ to the His-tag on the enzyme (Fig. S1†). The EziG carriers differ in their hydrophilicity as follows; opal is made without a polymer coating, resulting in a hydrophilic carrier, while coral is hydrophobic due to the addition of polyvinyl benzyl chloride on the carrier surface, and amber is a semi-hydrophilic carrier which has been blended with co-polymer.

In the case of both BlNahK and MtGlmU a similar degree of binding was observed for all the carriers trialled. The EziG affinity carriers and the amino carrier ECR8309F all showed binding capacities between 2–3.5 w/w% (Tables S2 and S3†). The exception to this was opal with BlNahK which had a higher degree of binding at approximately 5 w/w%. Initial indications therefore suggested that this was a suitable carrier for the immobilisation of BlNahK due to the higher loading.

**Fig. 2:**
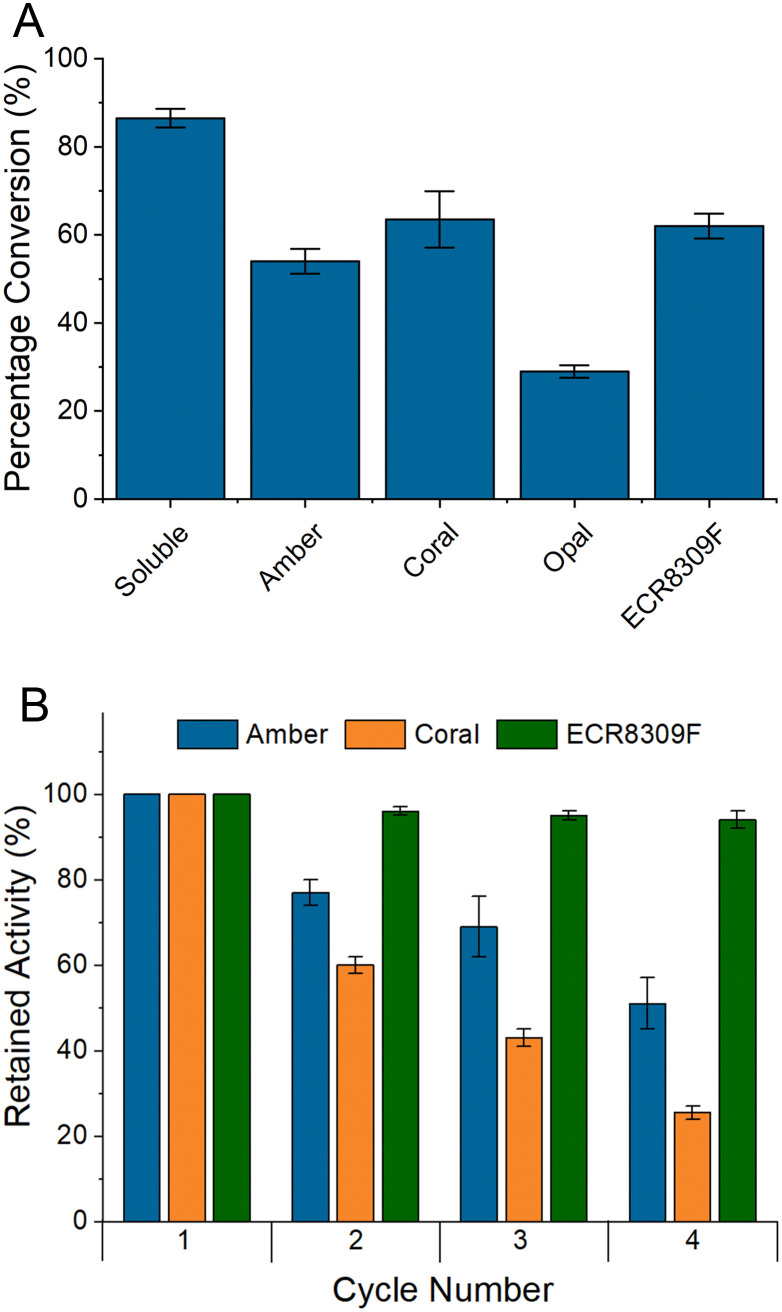
A Initial batch testing using BlNahK to form GlcNAc-1-P from GlcNAc. Showing immobilised enzymes in comparison to soluble enzyme as measured by ^1^H NMR. Masses of enzyme in each reaction; soluble 0.81 mg, amber 0.92 mg, coral 1.32 mg, opal 2.17 mg and ECR8309F 1.48 mg. B Further testing of successful carriers from the initial batch tests for four reaction cycles as measured by ^1^H NMR. Error bars are SEM, *n* = 2.

The results of small-scale batch reactions with BlNahK (Fig. 2A) demonstrated that while there was some loss in activity upon immobilisation, all but one of the carriers showed promise and could allow the reuse of the enzyme. Each carrier, except opal, was subsequently subjected to reuse in multiple consecutive reaction cycles (Fig. 2B), with a washing step in between. The lower activity of BlNahK on opal is surprising due to the higher loading; however, there could be multiple reasons for this observation. While more of the enzyme was initially bound, it is possible that this binding was weaker, leading to the enzyme being washed off in the steps prior to the reaction, or indeed that crowding/allosteric effects on the surface inhibited activity.^34^

The results (Fig. 2B) demonstrated that the most suitable resin was ECR8309F. Leaching of BlNahK from the affinity resins could be observed after each reaction cycle using Bradford reagent to observe protein in the supernatant, something which was not observed with the amino resin or in the case of other enzymes which were successfully bound to affinity resins.

**Fig. 3:**
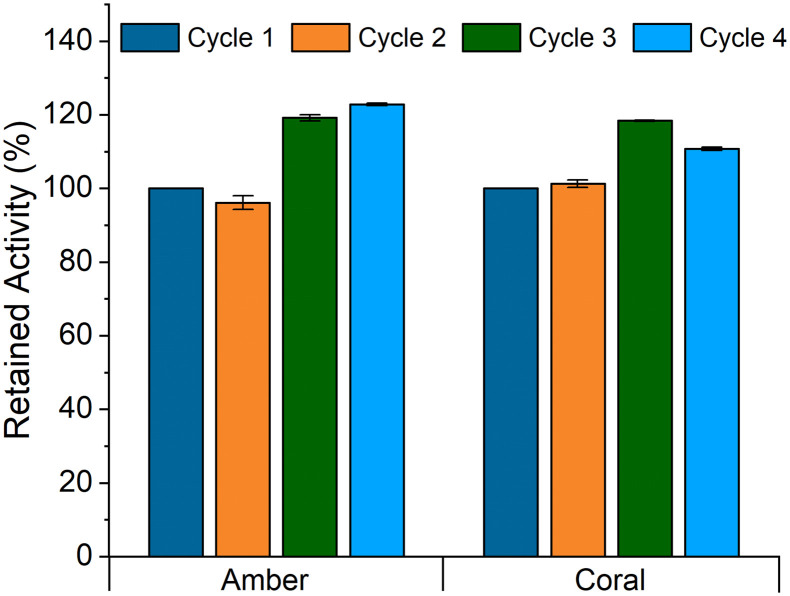
Batch testing of one pot reactions to yield UDP-GlcNAc from GlcNAc, using 0.5 mg mL^−1^ soluble BlNahK. Reactions incubated at 37 °C for 45 minutes, conversion was measured using integration analysis in ^1^H NMR. Initial conversion was 30% (set at 100% retained activity). Error bars are SEM, *n* = 2.

Initial immobilisation of MtGlmU was carried out solely on the affinity carriers due to the observation of stronger binding to a nickel column than the kinase, requiring 250 mM imidazole for elution during purification. Indeed, all three of the affinity carriers were observed to reach 100% conversion in just 45 minutes, justifying this approach. Enzyme leaching was not observed by Bradford test of the supernatant with coral or amber but was with opal, which may relate to the hydrophilic nature of opal. Due to the prohibitively high cost of GlcNAc-1-phosphate (GlcNAc-1-P), the recycling experiments were conducted as one-pot batch reactions consisting of soluble BlNahK together with one of coral or amber carriers to assess which was the most appropriate for continuous flow (to allow for *in situ* generation of GlcNAc-1-P). This would ensure any loss of retained activity could be attributed solely to the MtGlmU rather than a result of an immobilised BlNahK preparation losing activity across the reaction cycles and therefore lowering the concentration of GlcNAc-1-P available for the MtGlmU. Results from this batch testing (Fig. 3) demonstrated that both carriers had the potential to be utilised in continuous flow as both retained activity across four 45 minute reaction cycles (conversion was lower at 30% after 45 minutes using the immobilised preparation with soluble BlNahK). The apparent increase in activity could be attributed to a small quantity of soluble BlNahK binding to amber and coral and not being fully washed off by the washing steps (Fig. S2†). This increased effective concentration of BlNahK could have increased the rate of the initial biotransformation to GlcNAc-1-P providing immobilised MtGlmU with a higher substrate concentration, however the prohibitive cost of commercial GlcNAc-1-P necessitated this method.

When combining the results from both sets of reaction trials the strength of binding to the affinity carriers, unsurprisingly, directly correlated with the strength of binding to the nickel column. Similar results have been observed when immobilizing other enzymes within our lab, and as such we would suggest that for a metal affinity carrier to be a viable immobilisation method, binding to a nickel column should be strong, with a minimum of 100 mM imidazole required to elute the purified protein. Where less than 100 mM imidazole is required for elution, other methods such as covalent immobilisation would instead be recommended.

### Continuous flow

The immobilised preparations (BlNahK on 500 mg of ECR8309F and MtGlmU on 200 mg of coral) were transferred into individually packed columns to test under continuous flow conditions. UDP-GlcNAc was successfully produced, with both individual columns incubated at 37 °C and a flow rate of 45 μL min^−1^ affording a total residence time (*t*_res_) of 37 minutes, which is made up of a *t*_res_ of 22 minutes for the BlNahK reactor and 15 minutes for the MtGlmU reactor (system 3 – Table 1). The inequalities in the masses of the two carriers are a result of the type of binding to the carrier. As previously discussed, the ECR8309F carrier binds irreversibly in a non-selective manner, which is known to impact biocatalyst activity more than affinity binding. To account for this the mass of the non-selective immobilisation carrier was higher than for the selective EziG carrier. The EziG carriers, although binding in a reversible manner which is less stable, bind selectively to the His-tag, which generally has a lower impact on recovered activity. In the case of MtGlmU, the EziG preparation was bound strongly enough to allow for multiple reaction cycles while taking advantage of lower enzyme loadings. In addition, one of the key benefits of flow relevant to this process is the continuous removal of products, thus potentially minimising product inhibition effects.^15^ As such iPPase was not added to continuous flow reactions due to not needing to break down PPi, a known inhibitor of MtGlmU.^30^

**Table 1:** Continuous flow system used for UDP-GlcNAc production. Comparison of data between batch and flow processes. In run 1, both BlNahK and MtGlmU are soluble, whereas in runs 2–4 both enzymes are immobilised on their preferred carrier. (BlNahK; ECR8309F, MtGlmU: coral)

Run	Enzymes	Conditions	Flow rate (μL min^−1^)	Time	Reactor volume (mL)	Conv. (%)	STY (g L^−1^ h^−1^)
1	Soluble	Batch^a^	n/a	16.5 h	1	77	0.212
2	Immobilised	Batch^a^	n/a	48 h	100	95	0.096
3	Immobilised	Flow	45	37 min^b^	1.66^c^	30	2.37^d^
4	Immobilised	Flow	20	83 min^b^	1.66^c^	54	1.90^d^

a Batch reactions included 0.5 U mL^−1^ iPPase which was not required for continuous flow reactions.

b Total *T*_res_ combining both reactors for one full reactor volume.

c Total reactor volume, BlNahK bed volume: 0.99 mL MtGlmU bed volume: 0.67 mL.

d Calculated based on first full flow through, not accumulative reactions.

**Fig. 4:**
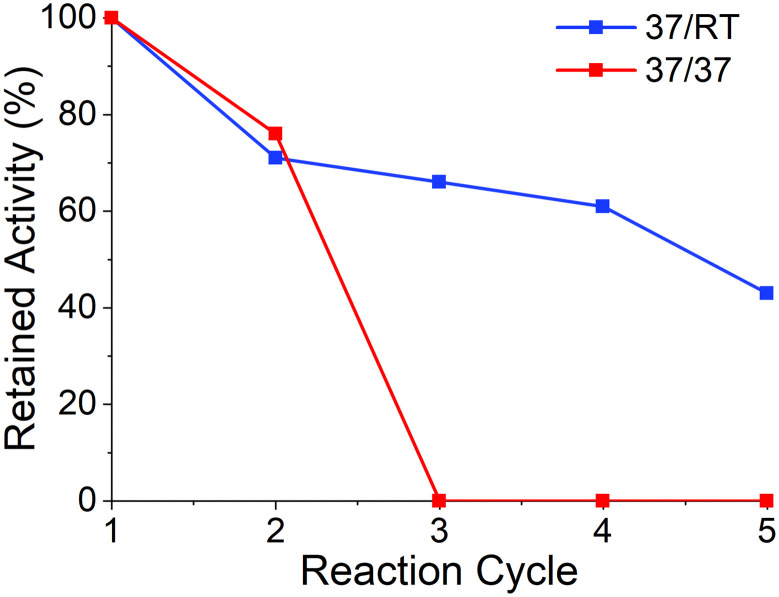
Retained activity across two flow systems where the temperatures were different for the two columns (37 °C and RT, blue line) and the same for the two columns (both 37 °C, red line).

After the first run, a steady state conversion of 30% was achieved across 10 reactor volumes. While the resulting STY for this system looked promising, the conversion was particularly low resulting in excess waste produced by the system. While a decrease in concentration could have improved this conversion, a decision was made to increase the *t*_res_ to allow for greater conversion, while taking a slight hit on the STY achieved. An increase in the *t*_res_ to 83 minutes resulted in a steady state conversion of 57% across 13 reactor volumes with both packed bed reactors held at 37 °C. To assess the reusability of the system, the columns containing each enzyme were placed in a storage buffer and stored at 4 °C before the reaction was run again. The results of the second cycle were promising, with a retained activity of 76%, however, a third run was conducted which showed a complete loss of activity of the system (Fig. 4, red line on graph). Upon more detailed analysis (Table S4†) it could be observed that the loss in activity of the system was predominantly due to a loss in activity of MtGlmU, while BlNahK retained a similar activity. It has been previously reported that another uridyltransferase enzyme, TaGalU, has a low thermal stability, and a decrease in reaction temperature yielded a more reusable immobilised biocatalyst.^35^

**Fig. 5:**
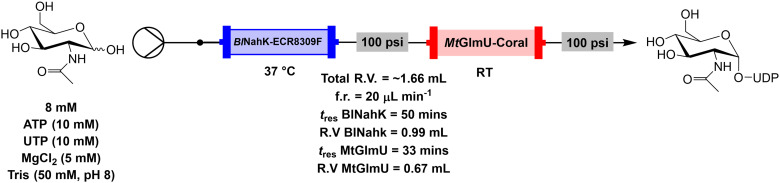
Continuous flow system set up for the biotransformation of GlcNAc to UDP-GlcNAc using immobilised enzymes in packed bed reactors.

We then exploited a key benefit of using a modular flow system to solve this problem. As each enzyme was loaded into separate packed bed reactors, different temperatures were maintained for each enzyme (Fig. 5). The reactions were repeated with MtGlmU at room temperature, while BlNahK was kept at 37 °C. It was observed that this change, while slightly lowering the conversion afforded in cycle one to a steady state conversion of 54%, allowed for a much greater reusability of the enzyme, with retained steady state activity maintained above 50% for four further reaction cycles (Fig. 4, blue line on graph, Fig. S3 and S4†).

To assess the benefits of our continuous flow approach to this cascade, we compared STY with a soluble batch reaction. As can be seen from the data presented in Table 1, the productivity for the continuous flow systems exceeded that of the soluble enzyme, with a greater potential for reuse obtained by the extension in biocatalyst lifetime afforded by the temperature difference in separate packed bed reactors. This, combined with the reusability of the system afforded by enzyme immobilisation, enables a scale up in the production of UDP-GlcNAc and potentially other UDP-sugars. When comparing time course experiments in batch, immobilisation of the cascade reduced activity of the enzymes but increased stability over time was observed (Table 1). While the lower activity results in prolonged reaction time, the increased stability gives rise to an increased biocatalyst lifetime and higher overall conversion (Fig. S5†).

## Conclusion

We have successfully produced UDP-GlcNAc on 100 mg scale under continuous conditions, with a steady state conversion of 54% ± 2% across twelve reactor volumes from an initial GlcNAc concentration of 8 mM. The system removed the need for a third enzyme to catalyse the breakdown of PPi by continuous by-product removal. The compartmental nature of the approach also allowed for the less thermally stable MtGlmU to be held in a separate packed bed reactor at a lower temperature, thus improving the reusability of the enzyme, and extending its lifetime for an extra three reaction cycles. This result demonstrates one of the key advantages of continuous flow systems above a repeated batch approach with either immobilised or soluble enzymes. Methods towards enzymatic glycosylation, whether that be of small molecules or proteins require reliable synthetic routes that yield substantial quantities of UDP-sugars. Due to the complex nature of chemical synthesis, enzymatic routes may prove a viable option for this large-scale approach. However, bioprocess development is required to enable this. As such, these results demonstrate a scalable method for the production of an important UDP-sugar from the sugar substrate. Applications towards glycosylation are anticipated, with impressive continuous demonstrations already shown by others highlighting the importance of UDP-sugar synthesis.^36^

## Data availability

The data supporting this article have been included as part of the ESI.†

## Conflicts of interest

There are no conflicts to declare.

## Supplementary Material

RE-010-D5RE00127G-s001
