# Supplementary material for: A modular, reusable biocatalytic flow system for UDP-GlcNAc production
Source: React Chem Eng. 2025 May 9;10(6):1221–6. doi: 10.1039/d5re00127g (PMC12079181; doi:10.1039/d5re00127g)
Supplement: RE-010-D5RE00127G-s001 [file RE-010-D5RE00127G-s001.pdf]

Supplementary Information (SI) for Reaction Chemistry & Engineering.
This journal is © The Royal Society of Chemistry 2025

A modular, reusable biocatalytic flow system for UDP-GlcNAc production

Tom L. Roberts,[1,2] Jonathan P. Dolan,[1,2] Gavin J. Miller,[*1,2] Marcelo A. D. Lima,[*2,3] Sebastian C. Cosgrove[*1,2]

1. Lennard-Jones Laboratory, School of Chemical & Physical Sciences, Keele University, Keele, Staffordshire, ST5 5BG (UK)
2. Centre for Glycoscience, Keele University, Keele, Staffordshire, ST5 5BG (UK)
3. School of Life Sciences, Keele University, Keele, Staffordshire, ST5 5BG (UK)

Email: s.cosgrove@keele.ac.uk; m.andrade.de.lima@keele.ac.uk; g.j.miller@keele.ac.uk;

Contents

Materials S2
Expression and purification S2
Enzyme immobilisation S2
Initial batch testing of kinases S3
Initial batch testing of uridylransferases enzymes S3
Soluble batch biocatalysis of UDP-sugars S3
Scaled up immobilised batch biocatalysis of UDP-sugars S3
Continuous flow biocatalysis of UDP-sugars S3

Materials

EziG resins were kindly supplied by EngineZyme (Stockholm, Sweden). Lifetech ECR8309F resin was kindly supplied by Purolite (Llantrissant, UK). Plasmid DNA was purchases from Biomatik (Canada). All other materials were supplied by Fisher Scientific (Loughborough, UK), ThermoFisher (Manchester, UK) or Biorad (Watford, UK).

Expression and purification

Plasmids were transformed into *E. coli* BL21 (DE3) chemically competent cells plated out on LB agar plates with the required antibiotic. A single colony was then picked, and a 10 ml LB starter-culture was incubated with required antibiotic overnight at 37 °C with shaking. This was grown up to either 1 L or 2 L using LB media, incubated with antibiotic at 37 °C with shaking until an optical density at 600 nm (OD600) of between 0.6 and 0.8 was achieved. Once the required OD600 was reached, expression was induced with Isopropyl ß-D-1-thiogalactopyranoside (IPTG) to a final concentration of 0.5 mM and the temperature cooled to 20 °C for 15 hours.

Resulting large scale culture was then centrifuged (22000 RCF, 45 minutes, 4 °C). The supernatant was discarded, and the cell pellet resuspended in appropriate volume bug buster according to manufacturer's instructions and DNase I at a working concentration of 0.05 mg mL^{-1}. This mixture was incubated with shaking (220 rpm, 45 minutes, RT) and then sonicated (60% power, 4 m 30 s, 2 s on, 3 s off). The resulting mixture was centrifuged (26895 RCF, 45 minutes, 4 °C) and the supernatant collected.

Enzymes were purified using Immobilised Metal Affinity Chromatography (IMAC) on a 5 mL HisTrap FF crude column, initially using a gradient elution method to determine optimum

imidazole concentration for elution of purified enzyme. Following this initial optimisation, once bound, the column was washed with 10 CV washing buffer (20 mM Tris-HCl, 0.5 M NaCl, 20 mM imidazole, pH 8). After washing, purified MtGlmU was eluted with elution buffer (20 mM Tris-HCl, 0.5 M NaCl, 250 mM imidazole, pH 8) or BlNahk was eluted with a separate elution buffer (20 mM Tris-HCl, 0.5 M NaCl, 100 mM imidazole, pH 8). Purified enzymes were analysed using SDS-PAGE (figure S6)
Purified enzymes were concentrated using a spin concentrator and then desalted into storage buffer (20 mM Tris-HCl, pH 8, 10% glycerol) using a PD-10 column. Once purified and desalted, enzyme concentration was estimated using a Nanodrop 1000, measuring the absorbance at 280 nm and calculating concentration using estimated molar absorption coefficients and molecular masses shown in table S1.

Enzyme immobilisation

EizG resins: 38 mg of the desired EziG supports were washed twice with washing buffer (200 mM Tris-HCl, pH 8). After washing, 1 mL of a known concentration of the purified enzyme solutions were mixed with the resin end over end for one hour at room temperature. After this the mixtures were centrifuged (15000 RPM, 1 minute), the supernatant extracted, and the resin washed twice with washing buffer. The supernatant was then analysed with a nanodrop 1000 to determine the concentration of the remaining enzyme. Equation 1 was then used to calculate the mass of enzyme immobilised on the resin:

Equation 1: Mass enzyme immobilised (mg) = mass offered (mg) - mass remaining (mg)

Purolite Lifetech ECR8309F: 50 mg of resin was washed with washing buffer and then incubated at room temperature with 4% glutaraldehyde solution for one hour. After further washing with washing buffer, the resin was incubated with purified BlNahk for 18 hours at room temperature.
For scaled up batch reactions and continuous flow reactions, BlNahk and MtGlmU were immobilised on 500 mg ECR8309F and 200 mg Coral respectively.

Batch testing of kinase

Soluble BlNahk was tested, in duplicate, in a batch reaction. BlNahk (0.8 mg mL^{-1}), sugar (8 mM), ATP (10 mM), and $MgCl_2$ (5 mM) were dissolved in reaction buffer (1 mL, 50 mM Tris-HCl, pH 8). The reaction mixture was then incubated with shaking at 37 °C for 45 minutes. Immobilised BlNahk was tested in a similar manner. Sugar (8 mM), ATP (10 mM), and $MgCl_2$ (5 mM) were dissolved in 1 mL of appropriate reaction buffer as above. The reaction mixture was then added to the immobilised biocatalyst prepared as described above and incubated with shaking at 37 °C for 45 minutes. Products were analysed using the characteristic anomeric hydrogen signals in ^1H NMR, compared against commercial standards.

Batch testing of uridylransferase

Soluble MtGlmU was also tested, in duplicate, in a batch reaction for comparison. BlNahk (0.5 mg mL^{-1}), MtGlmU (0.8 mg mL^{-1}), iPPase (0.5 U mL^{-1}), GlcNAc (8 mM), ATP (10 mM) UTP (10 mM), and $MgCl_2$ (5 mM) were dissolved in buffer (1 mL, 50 mM Tris-HCl, pH 8). The reaction mixture was then incubated with shaking at 37 °C for 45 minutes. Immobilised MtGlmU was tested in a similar manner. BlNahk (0.5 mg mL^{-1}), iPPase (0.5 U mL^{-1}), GlcNAc (8 mM), ATP (10 mM), UTP (10 mM) and $MgCl_2$ (5 mM) were dissolved in buffer (1 mL, 50 mM Tris-HCl,

pH 8). The reaction mixture was then added to the immobilised biocatalyst prepared as described above and incubated with shaking at 37 °C for 45 minutes.

Soluble batch biocatalysis of UDP-sugars

GlcNAc (8 mM), ATP (10 mM), UTP (10 mM) and $MgCl_2$ (5 mM) were dissolved in buffer (1 mL, 50 mM Tris-HCl, pH 8). BlNahk (1.64 mg mL^{-1}), MtGlmU (0.4 mg mL^{-1}) and iPPase (0.5 U mL^{-1}) were added and the reaction mixture was then incubated with shaking at 37 °C for 48 hours.

Scaled up immobilised batch biocatalysis of UDP-sugars

GlcNAc (8 mM), ATP (10 mM), UTP (10 mM) and $MgCl_2$ (5 mM) were dissolved in buffer (100 mL, 50 mM Tris-HCl, pH 8) and added to immobilised enzymes prepared as previously discussed together with the addition of iPPase (0.5 U mL^{-1}). Reaction mixture was incubated with shaking at 37 °C for 48 hours.

Continuous flow biocatalysis of UDP-sugars

GlcNAc (8 mM), ATP (10 mM), UTP (10 mM) and $MgCl_2$ (5 mM) were dissolved in buffer (25 mL, 50 mM Tris-HCl, pH 8). Immobilised enzymes were packed into glass Omnifit columns and connected in flow at a rate of 45 µL min^{-1} for system 3 and then 20 µL min^{-1} for system 4, collecting thirty-five fractions at a volume of 0.6 mL. Selected fractions were freeze dried, dissolved in D_2O and analysed using the characteristic anomeric hydrogen signals in ^1H NMR, compared against commercial standards. For system 3, the steady state percentage conversion was determined to be 30%, repeated cycles of at this flow rate were not completed due to the low percentage conversion. In the case of system 4 the steady state percentage conversion in the first reaction cycle was determined to be 54%. Retained activity remained over 60% for the first 4 reaction cycles.

Purifcation of UDP-sugars

UDP-GlcNAc produced from continuous flow biocatalysis was purified for structural determination. The reaction mixture was passed through a column packed with Bio-Gel P-2 resin (Bio-Rad) in 20 mM Ammonium formate to remove most of the reaction buffer. Sugar containing fractions were combined and subjected to three freeze dry cycles. The resulting mixture was then purified on an Agilent PL-SAX 1000 Å 150×25mm (PL1251-3102), using gradient elution of 0.1% formic acid in water up to 0.5M Triethylammonium bicarbonate (TEAB) buffer (pH 8.5). Residual TEAB was removed under vacuum. The resulting mixture was passed through a Na-exchange resin (Amberlite IRC-120, Na form). Finally, the mixture was passed through a column packed with Bio-Gel P-2 resin (Bio-Rad) in H_2O to yield purified UDP-GlcNAc sodium salt. (figure S7)

SDS-PAGE analysis of loaded affinity carrier

Carrier taken from reaction was washed with reaction buffer three times and then boiled using a heat block at 105 °C to release bound enzyme. The supernatant was then removed and analysed using SDS-PAGE providing evidence of BlNahK also binding to Coral during batch testing of MtGlmU.

NMR characterisation

^{1}H NMR (500 MHz, D_2O) δ 7.87 (d, J = 8.1 Hz, 1H), 5.93 – 5.85 (m, 2H), 5.43 (dd, J = 7.3, 3.3 Hz, 1H), 4.33 – 4.24 (m, 2H), 4.24 – 4.04 (m, 3H), 3.95 – 3.75 (m, 3H), 3.77 – 3.68 (m, 2H), 3.47 (dd, J = 10.1, 9.1 Hz, 1H), 1.99 (s, 3H). ^{13}C NMR (500 MHz, D_2O) δ 174.77, 166.23, 151.81, 141.65, 102.65, 94.49 (d, J = 6.32), 88.50, 83.20 (d, J = 9.2 Hz), 73.78, 73.01, 70.95, 69.58 (d, J = 13.1 Hz), 64.96 (d, J = 5.5 Hz), 60.33, 53.72, 53.63, 22.08. The data matches that reported in the literature.[1]

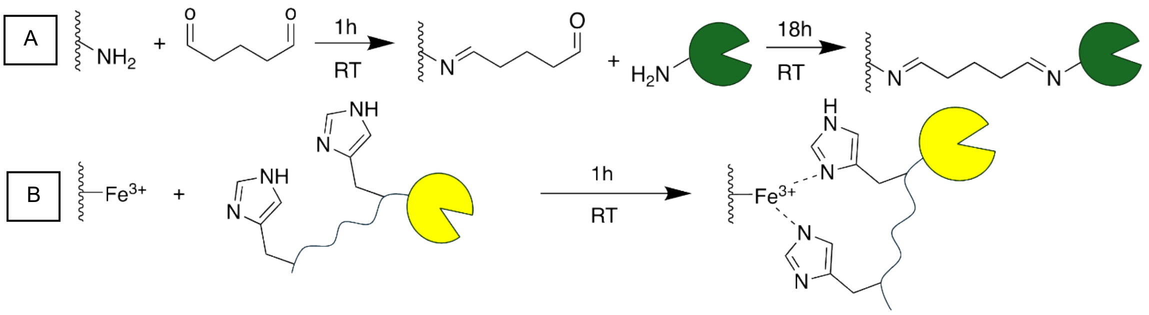

Figure S1 *Scheme showing the different immobilisation methods used in this study A; Covalent immobilisation to an amino resin, cross-linked with glutaraldehyde. B; Coordination of his-tag (two of six histidine residues shown) on enzyme to metal*

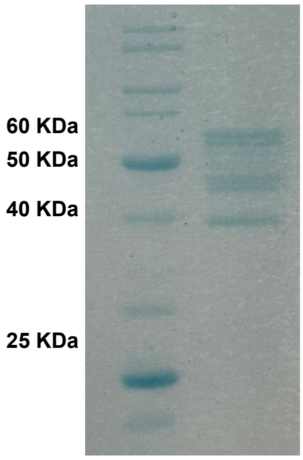

Figure S2 *SDS-PAGE analysis of Coral after initial batch testing of MtGlmU, showing BlNahK has also bound to resin during reactions.*

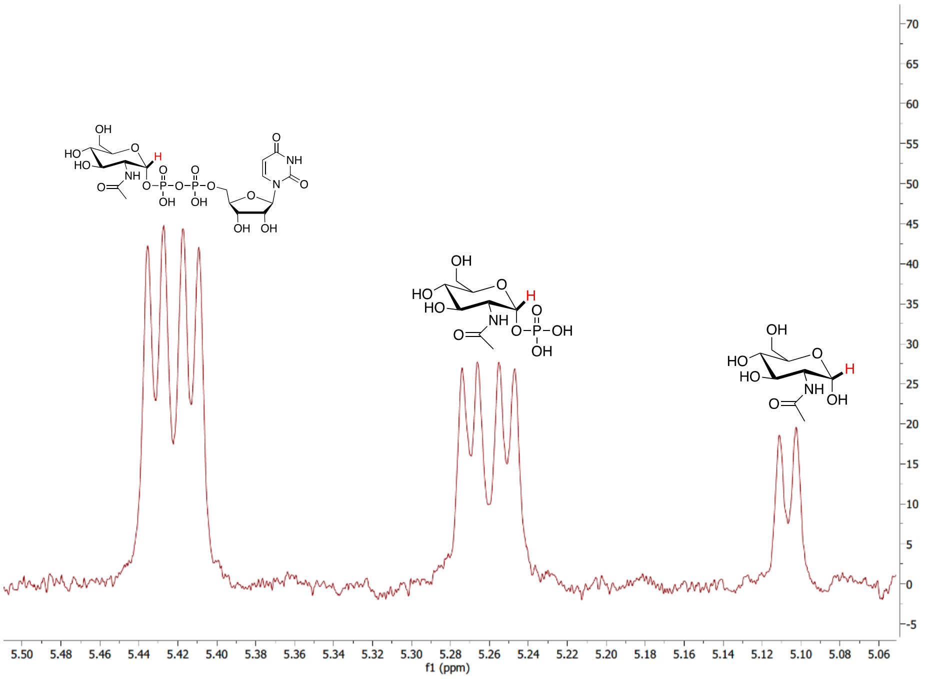

Figure S3 *¹H NMR spectra (500 MHz, D_2O) showing anomeric protons of UDP-GlcNAc, GlcNAc-1-P and GlcNAc, as observed in the first flow reaction system 4, integrations of which were used for the calculation of overall percentage conversion (54%).*

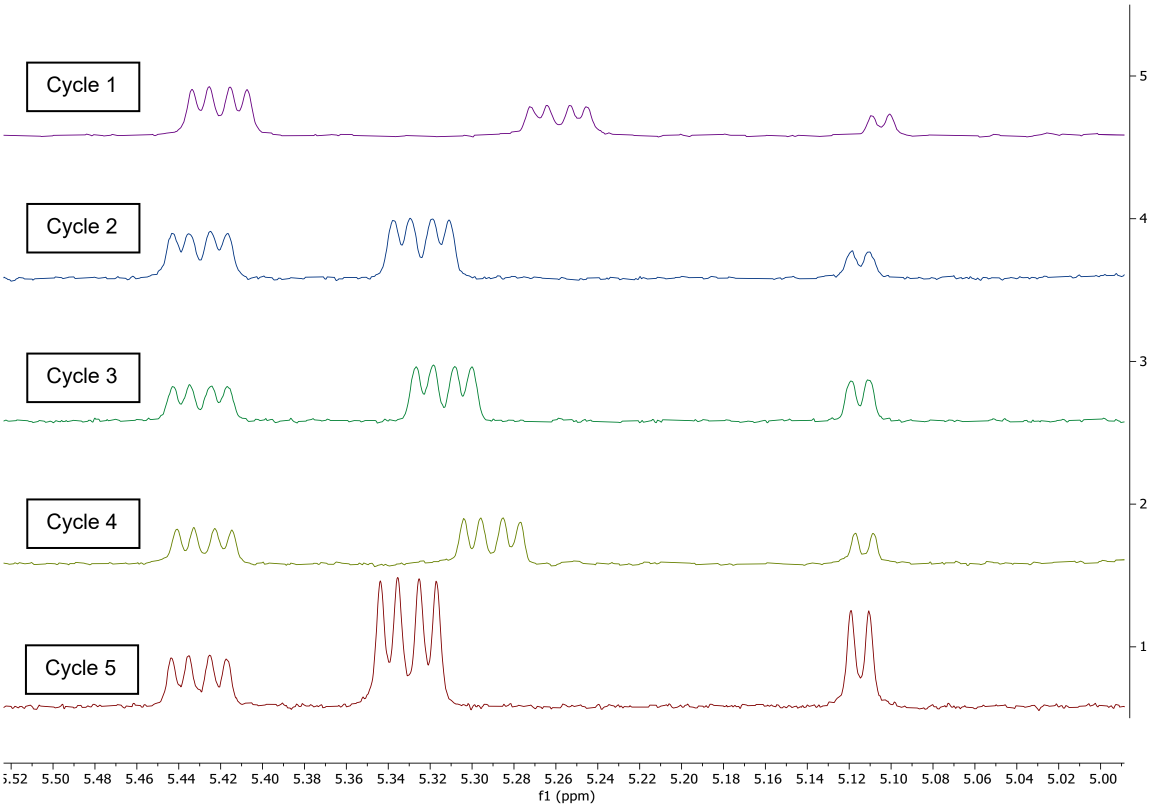

Figure S4 *¹H NMR spectra (500 MHz, D_2O) showing anomeric protons of UDP-GlcNAc, GlcNAc-1-P and GlcNAc, as observed across the five reaction cycles tested.*

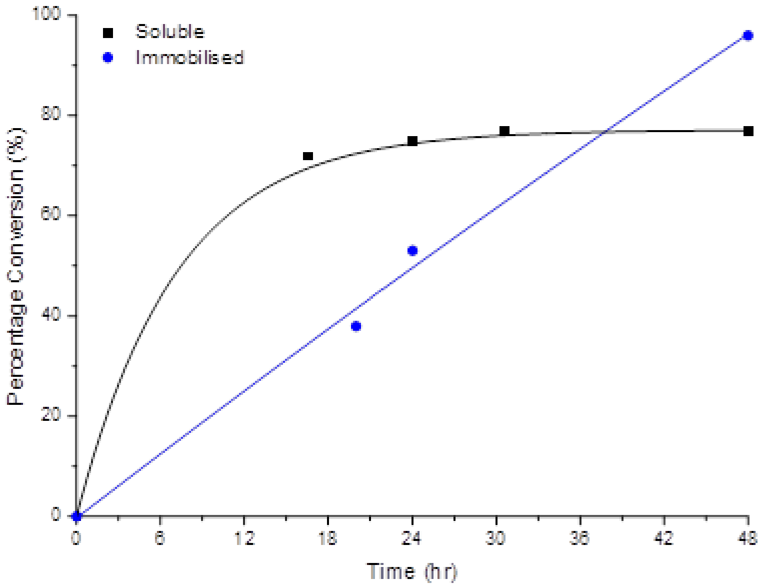

Figure S5 *Time course study of BlNahk and MtGlmU batch reactions.*

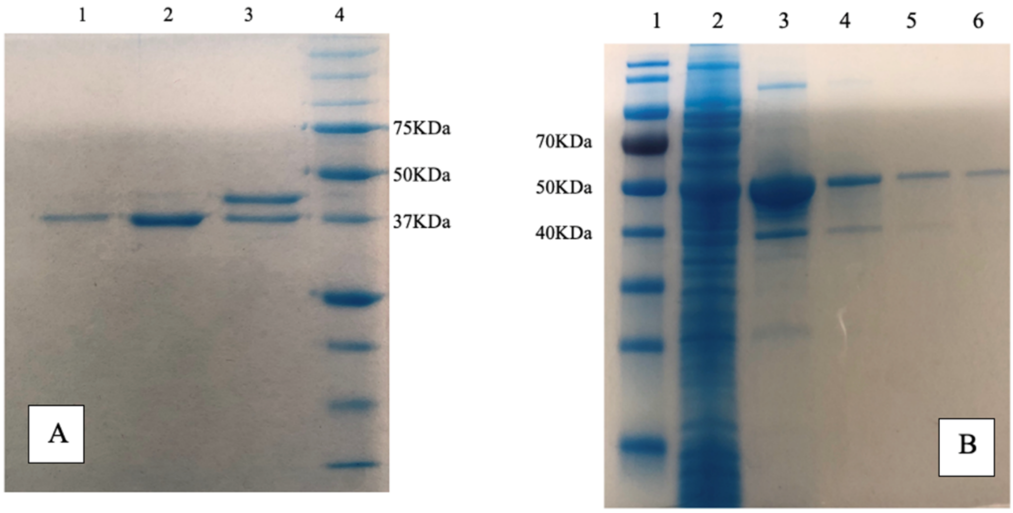

Figure S6 *SDS-PAGE gel of purified proteins. A Lane 1-3: Purified BlNahK,, Lane 4: SDS ladder. B: Lane 1: SDS ladder, Lane 2: lysate, lane 3-6 purified MtGlmU.*

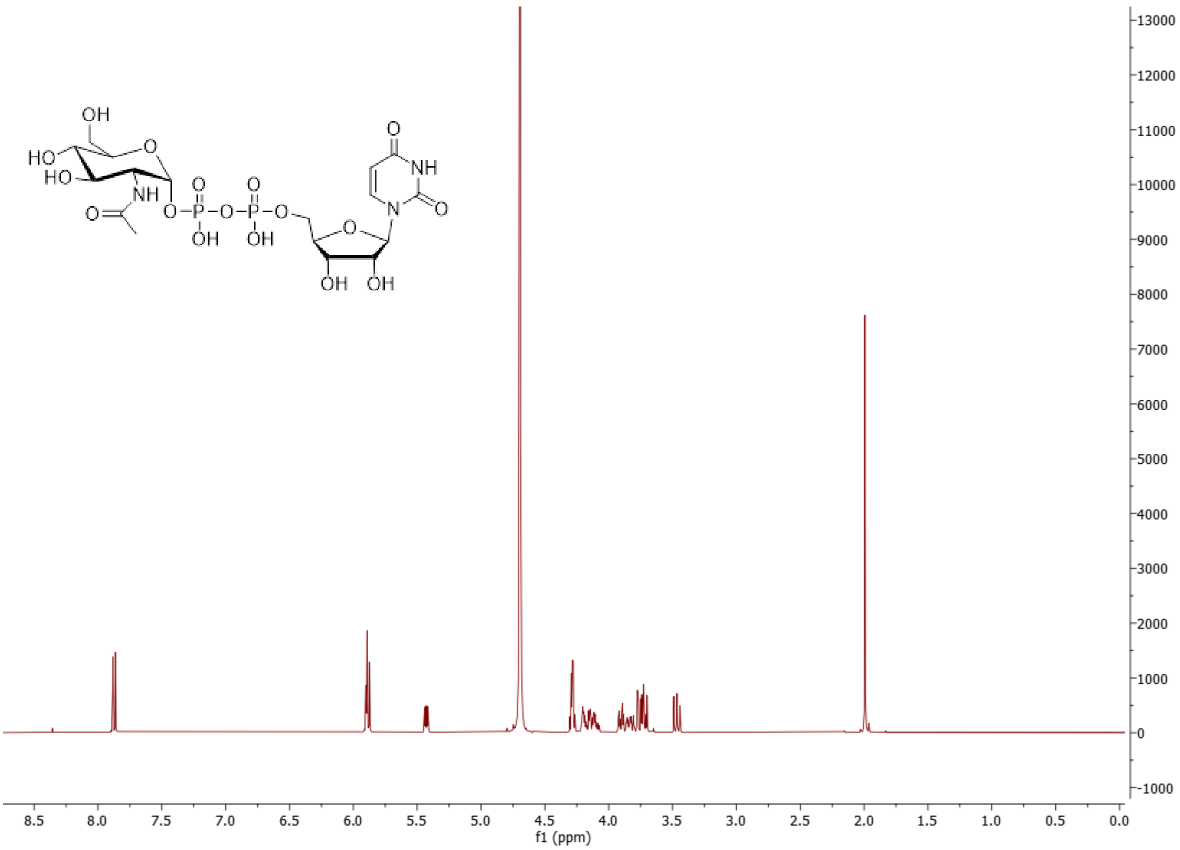

Figure S7 *^{1}H NMR spectra (500 MHz, D_2O) purified UDP-GlcNAc from continuous flow biocatalysis.*

Enzyme	Molar absorption coefficient (M^{-1} cm^{-1})	Molecular Mass (KDa)
BlNahk	27390	39.90
MtGlmU	24785	51.60

Table S1 *Molar absorption coefficients (ε) and molecular masses for enzymes in this study*

Experiment	Mass resin (mg)	Mass enzyme offered (mg)	Mass enzyme bound (mg)	w/w %	Immobilisation yield (%)
Resin trial Amber	38	2.66	0.92	2.35	35
Resin trial Coral	38	2.66	1.32	3.36	50
Resin trial Opal	38	2.66	2.17	5.41	82
Resin trial purolite	50	2.66	1.48	2.87	56
Flow/0.45 uL min^{-1} (purolite)	501	16.61	16.08	3.11	97
Flow/0.20 uL min^{-1} (purolite)	500	17.16	15.56	3.02	91
Flow/ 37/RT (purolite)	502	16.52	15.44	2.98	93

Table S2 *Immobilisation data showing masses of BlNahk immobilised on each carrier*

Experiment	Mass resin (mg)	Mass enzyme offered (mg)	Mass enzyme bound (mg)	w/w %	Immobilisation yield (%)
Resin trial Amber	38	2.21	1.18	3.01	53
Resin trial Coral	38	2.21	1.30	3.31	59
Resin trial Opal	38	2.21	0.83	2.13	38
Flow/0.45 uL min^{-1} (Coral)	199	9.36	8.13	3.93	87
Flow/0.20 uL min^{-1} (Coral)	200	9.36	6.52	3.16	70
Flow/ 37/RT (Coral)	200	8.84	6.36	3.08	72

Table S3 *Immobilisation data showing masses of MtGlmU immobilised on each carrier*

Cycle number	% GlcNAc	% GlcNAc-1-P	% UDP-GlcNAc
1	31	12	57
2	34	23	43
3	38	62	0

Table S4 *Analysis showing loss of activity due to loss of activity of MtGlmU in flow at 37 °C, while BlNahk retains similar activity across all three cycles.*

System	Conversion (%)	Substrate (mM)	Mass Nahk (mg)	Mass GlmU (mg)	Reactor volume (mL)	Total Tres (min)	Total reaction volume (mL)	Mass product (mg)	Reaction time (h)	STY (g L^{-1} h^{-1})	Total number of active cycles	Accumulated product across cycles (mg)
1	77	8	1.64	0.4	1	N/A	1	3.490	16.5	0.212	N/A	3.490
2	95	8	15.73	6.92	100	N/A	100	462	48	0.096	N/A	462
3	30	8	16.08	8.13	1.66	36.8	15.75	22.95	5.8	2.370	2	37.2
4	54	8	15.44	6.36	1.66	83	21	55.1	17.5	1.903	5	150

Table S5 *Analysis of the first cycle of different reaction systems utilised through this study*

References

1 S. Li, S. Wang, Y. Wang, J. Qu, X. Liu, P. G. Wang and J. Fang, *Green Chem.*, 2021, **23**, 2628–2633.
